# Matrix Stiffness Regulates Endothelial Cell Proliferation through Septin 9

**DOI:** 10.1371/journal.pone.0046889

**Published:** 2012-10-31

**Authors:** Yi-Ting Yeh, Sung Sik Hur, Joann Chang, Kuei-Chun Wang, Jeng-Jiann Chiu, Yi-Shuan Li, Shu Chien

**Affiliations:** 1 Department of Bioengineering, University of California San Diego, La Jolla, California, United States of America; 2 The Institute of Engineering in Medicine, University of California San Diego, La Jolla, California, United States of America; 3 Division of Medical Engineering Research, National Health Research Institutes, Zhunan, Taiwan; 4 Institute of Bioinformatics and Structural Biology and Department of Life Science, National Tsing-Hua University, Hsinchu, Taiwan; The University of Akron, United States of America

## Abstract

Endothelial proliferation, which is an important process in vascular homeostasis, can be regulated by the extracellular microenvironment. In this study we demonstrated that proliferation of endothelial cells (ECs) was enhanced on hydrogels with high stiffness (HSG, 21.5 kPa) in comparison to those with low stiffness (LSG, 1.72 kPa). ECs on HSG showed markedly prominent stress fibers and a higher RhoA activity than ECs on LSG. Blockade of RhoA attenuated stress fiber formation and proliferation of ECs on HSG, but had little effect on ECs on LSG; enhancement of RhoA had opposite effects. The phosphorylations of Src and Vav2, which are positive RhoA upstream effectors, were higher in ECs on HSG. The inhibition of Src/Vav2 attenuated the HSG-mediated RhoA activation and EC proliferation but exhibited nominal effects on ECs on LSG. Septin 9 (SEPT9), the negative upstream effector for RhoA, was significantly higher in ECs on LSG. The inhibition of SEPT9 increased RhoA activation, Src/Vav2 phosphorylations, and EC proliferation on LSG, but showed minor effects on ECs on HSG. We further demonstrated that the inactivation of integrin α_v_β_3_ caused an increase of *SEPT9* expression in ECs on HSG to attenuate Src/Vav2 phosphorylations and inhibit RhoA-dependent EC proliferation. These results demonstrate that the SEPT9/Src/Vav2/RhoA pathway constitutes an important molecular mechanism for the mechanical regulation of EC proliferation.

## Introduction

Many pathological conditions, such as vessel injury or tumor formation, alter the extracellular microenvironment for cells and influence cellular functions including proliferation and migration. It has been documented that the stiffness of arteries increases with the progression of vascular disease, hypertension, and diabetes [1]–[3]. In addition, tumor vasculature also exhibits a stiffer microenvironment [4]. Endothelial cells (ECs) lining the vessel wall control vascular permeability and homeostasis, and their dysfunction, such as inflammation and proliferation, may lead to the development of vascular diseases and tumor angiogenesis. Changes of the extracellular matrix (ECM) stiffness can influence EC functions [5], [6]. On soft collagen gels ECs show capillary morphogenesis, whereas on stiff gels the EC capillary network decreases [7] and spreading area increases [8]. EC spreading and focal adhesion formation are attenuated in ECs co-cultured with smooth muscle cells (SMCs, with the SMC layer serving as a soft substrate) and in ECs seeded on soft polyacrylamide gels in comparison to ECs on hard surfaces [9]. Such EC-SMC co-cultures serve as a model of the healthy vessel wall to inhibit the TNF-α-induced EC inflammation [10]. Moreover, ECs derived from tumor stromas with high stiffness show distinct differences in structure, signaling, and function when compared with ECs from normal tissues [11]. While these studies demonstrate that ECM mechanics play a pivotal role in regulating EC functions, the molecular mechanisms by which ECs respond to the altered ECM mechanics to regulate cell proliferation remain to be elucidated.

Cells respond to changes in their mechanical surroundings (e.g., alterations of substrate stiffness) by reorganizing their cytoskeleton, thus adjusting the contractile force exerted on the substrate and modifying their biological and mechanical behaviors [12]. The adherent cells sense the environmental changes through the ECM-integrin interactions to transmit structural and biochemical signals, and such outside-in signals activate a Rho-GTPase-modulated feedback loop. This in turn regulates intracellular contraction to generate force (inside-out) through the cytoskeleton to further enhance the integrin clustering and focal adhesion assembly. The balance of the outside-in and inside-out signaling leads to the regulation of adhesion-mediated cell functions such as proliferation [13], [14].

Rho-GTPase, a major player for cytoskeleton dynamics and cell contractility (cellular tension), is positively regulated by guanine nucleotide exchange factors (GEFs) and negatively regulated by GTPase activating proteins (GAPs) [15]. It has been reported that ECM stiffness can alter the cellular force balance and in turn activate Rho-mediated contractility (tension) to enhance tumor growth [14]. Src has also been reported to be an important regulator in modulating GEF and GAP activities [16]. Under mechanical stretch, Src activation causes the phosphorylation of Vav2, a ubiquitously expressed GEF, to increase the Rho-GTPase activity in mesangial cells [17]. A dominant negative mutant of Vav2 attenuates Rho-GTPase activity and blocks stress fiber formation in MDCK cells [18]. Septin 9 (SEPT9) is reported to associate with p114GEF to inhibit Rho-GTPase activity in growing REF cells [19]. Septins are a family of GTP-binding proteins that assemble into filaments to constitute a non-canonical cytoskeleton [20] and have been shown to regulate cortical actin formation and hence, the cortical rigidity of cells [21]. SEPT9 perturbations have been reported in some cases of breast and ovarian cancers, implying that SEPT9 plays a role in regulating cell proliferation and may be a candidate tumor suppressor gene [22], [23]. The role of SEPT9 in endothelial mechanotransduction and proliferation, however, has yet to be determined.

In the present study, we identified a novel role of SEPT9 in regulating EC proliferation on substrates with different stiffnesses. We demonstrated that the expression of SEPT9 is increased in ECs cultured on the low-stiffness substrate (LSG) that leads to the attenuation of Src/Vav2 phosphorylations and the inhibition of RhoA-dependent cell cycle progression. These results reveal an important molecular mechanism for the mechanical regulation of EC proliferation.

## Results

### Substrate stiffness modulates stress fiber distribution and cell proliferation in ECs

**Figure 1:**
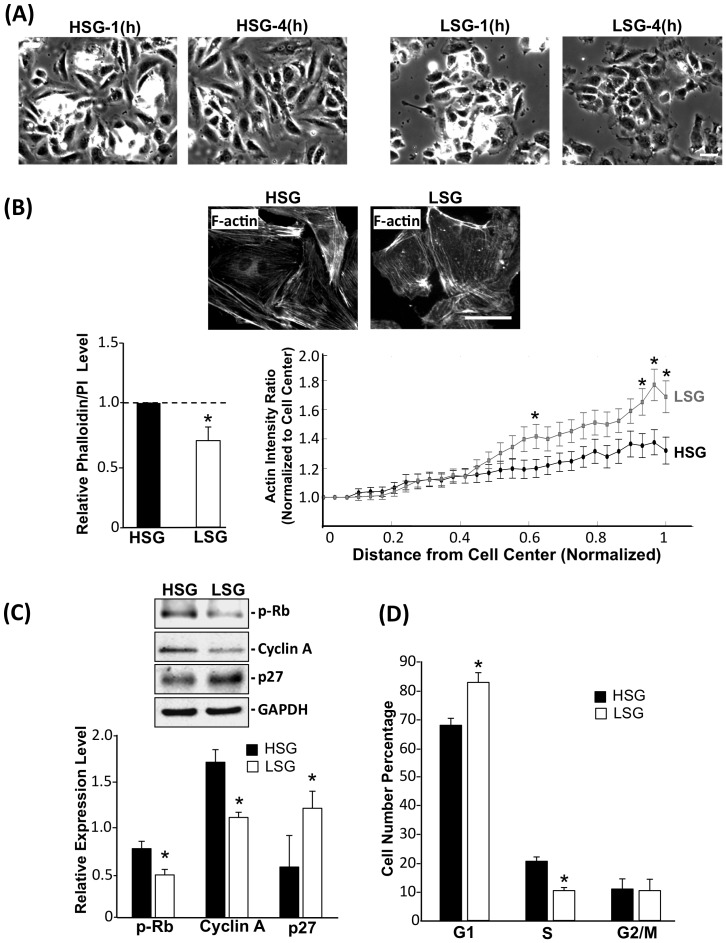
Substrate stiffness regulates cytoskeletal organization and EC proliferation. (A) Representative phase images showing the morphology of ECs seeded and allowed to spread on HSG and LSG for the indicated time periods. Scale bar = 50 µm. (B) F-actin staining with FITC-labeled phalloidin. The line graph (right lower panel of B) shows the normalized radial distribution of scanned fluorescent intensity from cell nucleus to cell boundary, n = 24 cells. * p<0.05 for comparison between HSG and LSG for each normalized radial position. Bar graph (left panel of B) shows the F-actin fluorescence intensities for HSG and LSG normalized with cell numbers. * p<0.05 for comparison between HSG and LSG. Scale bar = 50 µm. (C) Immunoblotting with antibodies against hyper-phosphorylated Rb, cyclin A, and p27 for ECs lysed after seeded on HSG and LSG for 24 h. Bar graph represents the quantified results of three independent experiments. * p<0.05 for comparison between HSG and LSG. (D) Cell cycle populations were analyzed by flow cytometry. * p<0.05 between HSG and LSG (n = 3).

It has been reported that the matrix stiffness is higher in injured vessels compared with normal and that the increase of stiffness correlates well with cell proliferation [24]. To understand how extracellular environmental cues contribute to EC proliferation, we used polyacrylamide (PA) gels with different degrees of stiffness as cell adhesion/growth substrates to mimic the physiological and pathological conditions [5], [24]–[26]. As shown in Fig. S1, HUVECs were cultured on glass (>60 GPa) and substrates with high (21.5 kPa), medium (9.7 kPa) and low stiffness (1.72 kPa). The resulting EC proliferation decreased as stiffness decreases; statistic analyses demonstrated that no significant difference between the ECs on glass and substrate of 21.5 kPa. These results suggest that 21.5 kPa may have already reached the plateau for substrate stiffness-mediated EC proliferation. ECs were cultured on high-stiffness gel (HSG, 21.5 kPa) and low-stiffness gel (LSG, 1.72 kPa) (Table 1) for various time durations. The images of ECs on HSG and LSG were acquired using an inverted fluorescence microscope (Fig. 1A). As shown in Fig. 1A, cells seeded on HSG showed spindle-like and well-spread morphology, whereas cells on LSG showed a round shape and smaller size. To quantify the actin level in ECs on HSG and LSG, total phalloidin fluorescence staining intensities in the field were normalized against the cell numbers determined by staining with propidium iodide (PI). The results showed that ECs on LSG exhibited a significantly lower actin level than on HSG (Fig. 1B, left panel). The radial distribution of actin in a cell was determined by radial scans taken every 10° (from 0° to 360°). Each radial scan within a cell was partitioned into 30 fractions from cell center to the edge, and the fluorescence intensity averaged at each fractional distance across the cell (Fig. S2). The results showed that the actin level was evenly distributed across the cell body of ECs on HSG (Fig. 1B right panel) and that a preferential distribution of actin in the peripheral cortical layer was observed in ECs on LSG (Fig. 1B, right panel). To assess the effects of different substrate stiffness on EC functions, cell cycle regulatory proteins and EC proliferation were analyzed. ECs cultured on LSG expressed significantly lower levels of cell cycle promoting regulators, such as cyclin A and hyper-phosphorylated Rb, with a significantly higher level of the cell cycle suppressor protein p27, in comparison with ECs on HSG (Fig. 1C). Flow cytometry analyses showed an increase of G1-phase cells and a decrease of S-phase cells on LSG in comparison to cells on HSG (Fig. 1D) indicating that cell cycle progression is enhanced on HSG in comparison with LSG. These results suggest that substrate stiffness can alter cell shape by changing the cytoskeleton structure and modulate cell proliferation through the regulation of cell cycle-related proteins.

**Table 1:** Characteristics of polyacrylamide hydrogels.

Sample Name	Final Acry%	Final Bis%	Young's modulus (kPa)
**High Stiff-Gel (HSG)**	10	0.2	21.5±0.06
**Low Stiff-Gel (LSG)**	5	0.05	1.72±0.20

### RhoA activity and stress fibers are required for stiffness-regulated EC proliferation

**Figure 2:**
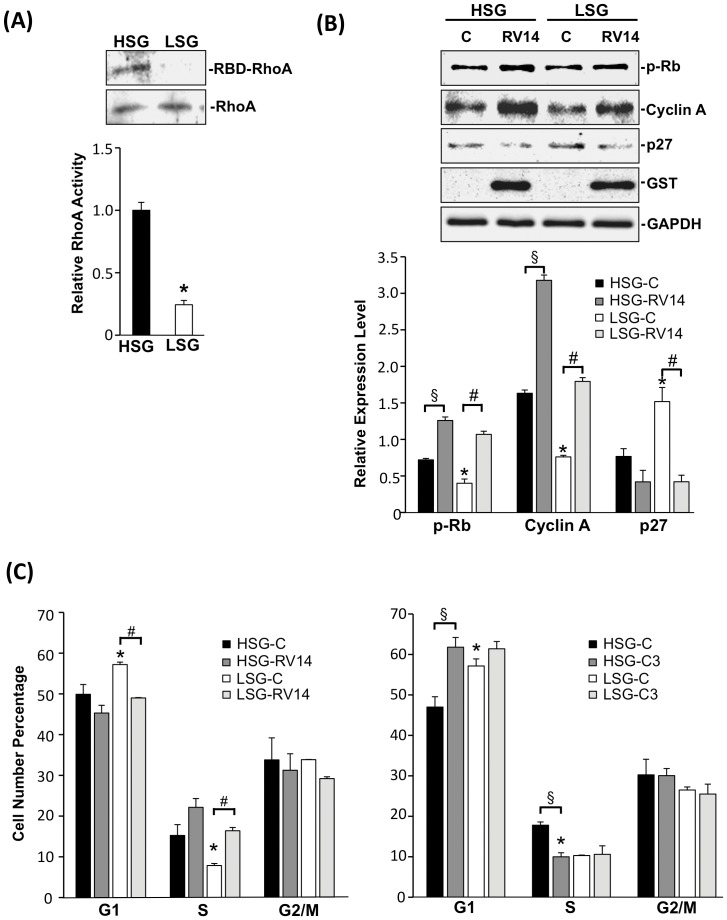
RhoA mediates the substrate stiffness-regulation of EC proliferation. (A) ECs were seeded on HSG and LSG for 24 h and cell lysates were subjected to RBD pull down assay. Immunoblotting of active RhoA (RBD-RhoA) and total RhoA was performed by using an antibody against RhoA. * p<0.05 for comparison between HSG and LSG (n = 3). (B) ECs were transfected with empty vector or GST-tagged RhoV14 (RV14). 24 h post-transfection, cells were seeded on HSG and LSG for another 24 h. Immunoblotting of cell cycle regulatory proteins was performed by using antibodies against hyper-phosphorylated Rb, cyclin A, and p27. * p<0.05 in comparison with the corresponding controls on HSG and LSG. § p<0.05 for comparison between control and RV14 on HSG. # p<0.05 for comparison between control and RV14 on LSG (n = 3). (C) Cells were transfected with the empty vector or RV14 (left panel of C) and empty vector or C3 (right panel of C). Bar graphs of flow cytometry analyses show the percentages of cells in G0/G1, S, and G2/M phases of ECs on HSG and LSG. * p<0.05 in comparison with the corresponding controls on HSG and LSG. # p<0.05 for comparison between the control and RV14 on LSG. § p<0.05 for comparison between the control and C3 on HSG (n = 3).

RhoA is reported to mediate shape-dependent cell growth [27]. To elucidate the role of Rho-GTPase in stiffness-regulated cell proliferation, RhoA activity was measured in ECs on substrates with different stiffnesses using the RBD-pull down assay. ECs on LSG exhibited a significantly lower activity of RhoA than those on HSG (Fig. 2A). To further investigate the involvement of RhoA activity in stiffness-regulated cell growth, the constitutively active form of RhoA (RV14) protein with GST tag was transfected into ECs. RV14 caused increases of cyclin A expression and Rb hyper-phosphorylation on both HSG and LSG and a significant reduction of p27 expression on LSG (Fig. 2B). RV14 caused a decrease of G1-phase cells and an increase of S-phase cells in ECs on LSG but had no significant effect on ECs on HSG. C3 exoenzyme, which inactivates RhoA, caused an increase in G1-phase cells and a decrease in S-phase cells in ECs on HSG but had no significant effect on ECs on LSG (Fig. 2C). In concert with the well-known role of Rho-GTPase in stress fiber regulation [28], we found that EC stress fiber formation was enhanced by RV14 and reduced by C3 exoenzyme (Fig. S3). These results indicate that RhoA activity and the consequent modulation of actin distribution may play an important role in the stiffness-regulation of cell proliferation.

### HSG-induced EC proliferation is mediated through Src/Vav2/RhoA pathway

**Figure 3:**
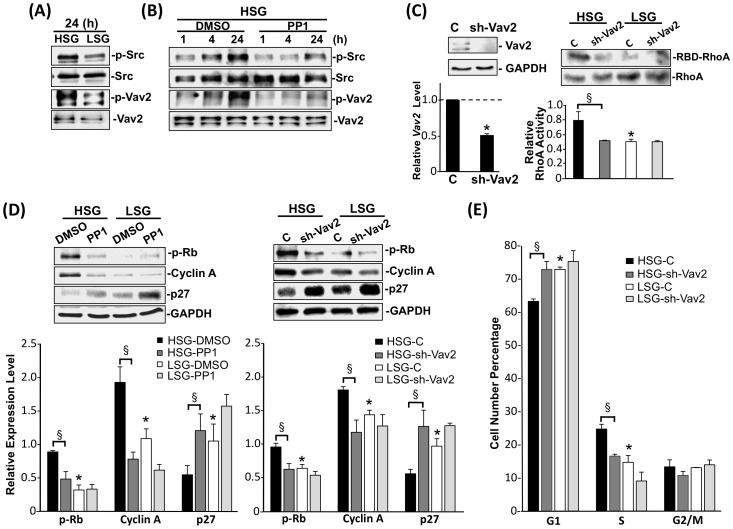
HSG modulates EC proliferation through the Src/Vav2 pathway. (A) ECs were seeded on HSG and LSG for 24 h, and cell lysates were immunoblotted with antibodies against phospho-Src (Y416), Src, phospho-Vav2 (Y172), and Vav2. (B) ECs pretreated with DMSO and PP1 (20 µM) for 1 h were seeded on HSG for 1 h, 4 h or 24 h. Cell lysates were analyzed for phospho-Src (Y416), Src, phospho-Vav2 (Y172), and Vav2. (C) Vav2 was knocked down in ECs by shRNA (sh-Vav2) with more than 50% reduction in Vav2 RNA and protein levels (left lower and upper panels, respectively). Cells with sh-Vav2 and control-shRNA were seeded on HSG and LSG for 24 h, and cell lysates were subjected to an RBD-pull down assay with an antibody against RhoA (right panel). (D–E) ECs were pretreated with PP1 or transfected with Vav2-shRNA (sh-Vav2), seeded on HSG and LSG for 24 h, and subjected to immunoblotting for protein expression (D) or flow cytometry for cell cycle analyses (E). * p<0.05 in comparison with the corresponding controls on HSG and LSG. § p<0.05 for comparison between DMSO and PP1 or between control and sh-Vav2 on HSG (n = 3).

To assess the mechanisms by which ECs respond to substrate stiffness, we investigated the kinase activities in EC focal adhesion complexes. Src has been reported to phosphorylate the tyrosine-172 of Vav2, a RhoGEF, to modulate Rho-GTPase activities [15], [17]. As shown in Fig. 3A, HSG induced higher levels of Src and Vav2 phosphorylations in ECs than LSG. Pretreatment with a Src kinase inhibitor, PP1 (20 µM), inhibited the HSG-induced Src and Vav2 phosphorylations, indicating that Src is the upstream kinase for Vav2 phosphorylation on HSG (Fig. 3B). The knockdown of Vav2 expression by transfecting the Vav2-shRNA plasmid into ECs reduced the RhoA activity in ECs on HSG to the level seen on LSG (Fig. 3C), but had no effect on Src phosphorylation (data not shown). In ECs on HSG, the inhibition of Src (with PP1) and Vav2 (with sh-Vav2) also significantly lowered the cyclin A expression and Rb hyper-phosphorylation while augmenting p27 expression, in comparison with corresponding controls (Fig. 3D). Src inhibition by using the Src specific-siRNA also had the same effects as PP1 treatment in ECs on HSG (Fig. S4). In addition, the knockdown of Vav2 increased the G1-phase cells and decreased S-phase cells in ECs on HSG (Fig. 3E). These results indicate that the HSG activates the Src/Vav2 pathway to enhance cell proliferation compared to ECs on LSG.

### SEPT9 mediates the RhoA inhibition and cytoskeleton distribution in ECs on LSG

**Figure 4:**
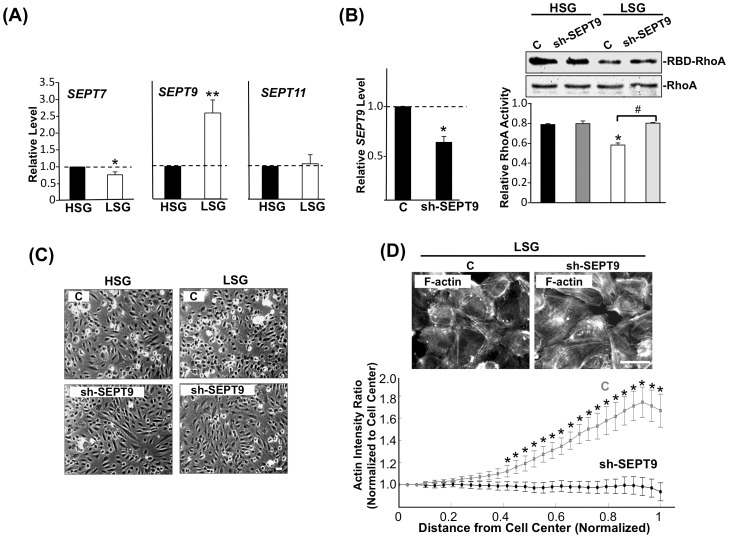
LSG-induced *SEPT9* expression inhibits RhoA activity and peripheral actin distribution. (A) Gene expression profiles for *SEPT7*, *SEPT9*, and *SEPT11* in ECs cultured on LSG and HSG for 24 h. Data represent mean ± SEM (Student's t-test; * p<0.05, ** p<0.01). (B) Gene expression profile of *SEPT9* after transfection with sh-SEPT9 (left panel). RhoA activity level after transfection with sh-SEPT9 was followed by RBD-pull down assay (right panel). * p<0.05 in comparison with the corresponding controls on HSG and LSG. # p<0.05 for comparison between control and sh-SEPT9 on LSG. (n = 3). (C) Phase microscopy images of ECs seeded on HSG or LSG for 24 h in response to sh-SEPT9 transfection. Scale bar = 50 µm. (D) ECs with control and sh-SEPT9 transfection were seeded on LSG for 3 h. Actin was stained with FITC-phalloidin and the cellular distribution was quantified by image analysis using the intensity ratio profile program (Fig. S1). * p<0.05 for comparison between control and sh-SEPT9 at successive normalized radial positions from the cell center (0) to the periphery (1). n = 17 cells. Scale bar = 50 µm.

As shown in Fig. 1B, ECs on LSG exhibited greater cortical actin distribution and a lower RhoA activity than the ECs on HSG. Septin (SEPT) family proteins are highly correlated with cortical actin integrity [21] and RhoA activity inhibition [19]. Actin stress fibers have been shown to colocalize with the SEPT7-SEPT9-SEPT11 polymer complex and are functionally and structurally interdependent [29]. As shown in Fig. 4A, *SEPT9* expression was markedly higher in ECs on LSG in comparison to HSG. The expression of *SEPT7* was slightly lower in ECs on LSG, whereas *SEPT11* expression did not differ significantly between HSG and LSG. To test whether SEPT9 is involved in regulating the differences in morphology and cytoskeletal distribution in ECs on HSG and LSG (Figs. 1A and B), we inhibited SEPT9 expression using the SEPT9-shRNA plasmid. Transfection of the SEPT9-shRNA plasmid resulted in ∼40% reduction of *SEPT9* expression level in cells in comparison to the control-shRNA cells (Fig. 4B; left panel). Inhibition of SEPT9 caused ECs to become more elongated and spindle shaped on both LSG and HSG (Fig. 4C). The knockdown of SEPT9 also caused a significant increase in RhoA activity (Fig. 4B; right panel) and a remodeling of actin distribution from the peripheral to the central region in ECs on LSG (Fig. 4D). These results indicate that SEPT9 mediates the stiffness-regulation of RhoA activity and cortical actin distribution.

### SEPT9 regulates RhoA-mediated EC proliferation through p114GEF and the Src/Vav2 pathway

**Figure 5:**
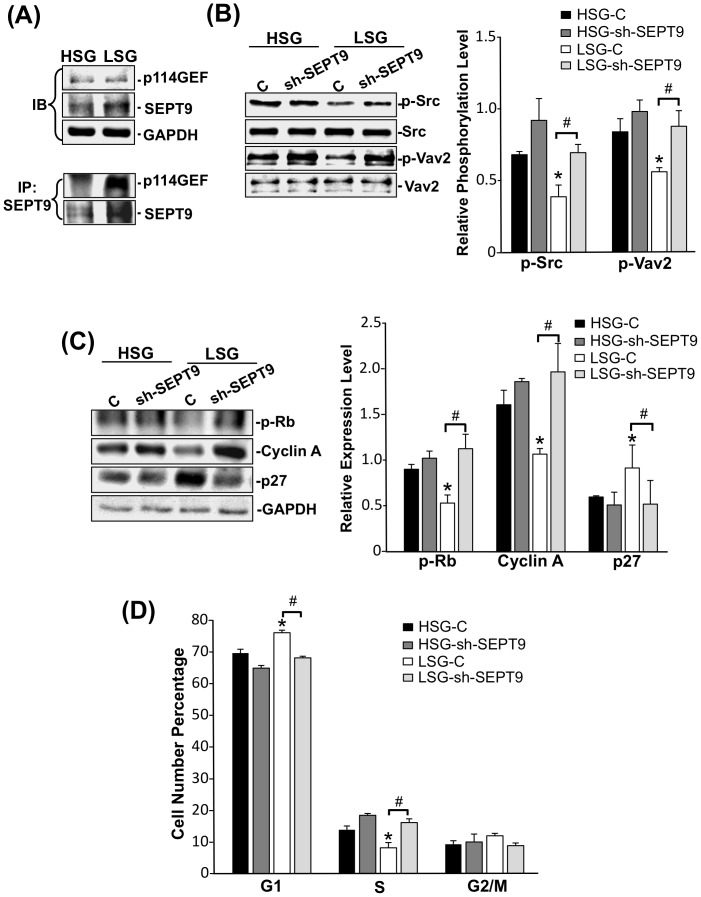
SEPT9 regulates p114GEF and Src/Vav2/RhoA pathway to modulate EC proliferation. ECs were seeded on HSG and LSG for 24 h. (A) The total cell lysates were immunoblotted with antibodies against p114GEF and SEPT9 (upper panel) and immunoprecipitated with an antibody against SEPT9 followed by immunoblotting with antibodies against p114GEF and SEPT9 (lower panel). (B–C) ECs transfected with control-shRNA or sh-SEPT9 were seeded on HSG and LSG for 24 h, and the cell lysates were immunoblotted with antibodies against phospho-Src (Y416), Src, phospho-Vav2 (Y172), and Vav2 (B) and hyper-phosphorylated Rb, cyclin A, and p27 (C). Bar graph represents the quantified results of three independent experiments. (D) ECs transfected with control-shRNA or sh-SEPT9 were subjected to flow cytometry for cell cycle analyses. * p<0.05 in comparison with the corresponding controls on HSG and LSG. # p<0.05 for comparison between control and sh-SEPT9 on LSG (n = 3).

Our results showed that ECs on LSG expressed a higher level of SEPT9 that leads to the inhibition of RhoA (Fig. 4B; right panel). SEPT9 has been shown to function as a RhoA inhibitor by associating with the p114GEF in REF cells [19]. We observed that the SEPT9-associated p114GEF was increased in ECs on LSG compared with ECs on HSG (Fig. 5A) suggesting that the increase in SEPT9 expression on LSG enhances the recruitment of p114GEF into the complex, which in turn reduces the RhoA activation. Our results showed that the Src/Vav2 pathway modulated RhoA activity in ECs on substrates with different stiffnesses (Fig. 3). Inhibition of SEPT9 increased both Src and Vav2 phosphorylations in ECs on LSG (Fig. 5B), suggesting that SEPT9 regulates RhoA activity not only by recruiting p114GEF but also by inhibiting the Src/Vav2 pathway in ECs on LSG. To further investigate the role of SEPT9 in regulating EC functions, we found that the knockdown of SEPT9 significantly increased the levels of cyclin A expression and Rb hyper-phosphorylation and decreased the expression level of p27 in ECs on LSG (Fig. 5C). These changes were accompanied by a decrease in G1-phase cells and an increase in S-phase cells in ECs on LSG (Fig. 5D). The above results indicate that SEPT9 plays a critical role in substrate stiffness-regulation of signaling which leads to morphological and functional consequences.

### Integrin α_v_β_3_ mediates stiffness-modulation of *SEPT9* expression and EC proliferation

**Figure 6:**
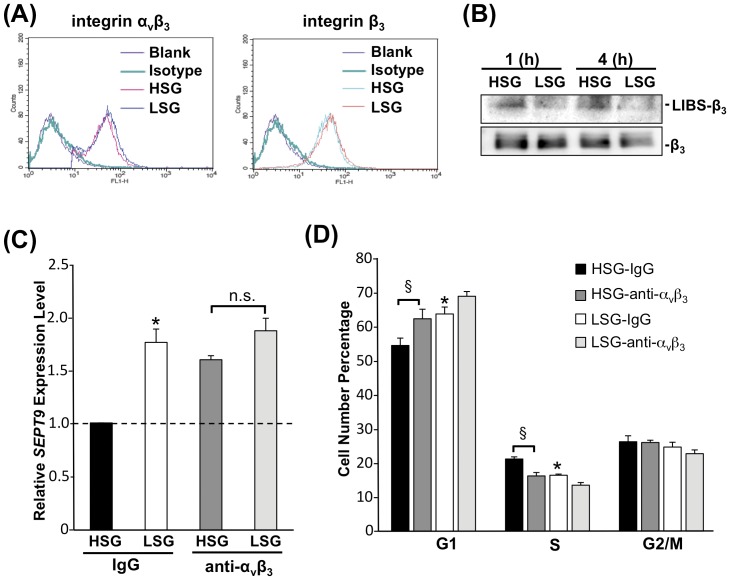
Integrin α_v_β_3_ mediates the stiffness-modulation of *SEPT9* expression and EC proliferation. (A) Integrin expression profiles of ECs on HSG and LSG. ECs on HSG and LSG for 24 h were stained with integrin antibodies against integrin α_v_β_3_ and integrin β_3_ and analyzed by flow cytometry. The green-line histogram represents the isotype control. The red- and blue-line histograms represent the surface expressions of the specific integrins on HSG and LSG, respectively. (B) ECs were seeded on HSG and LSG for the indicated times and cell lysates were immunoblotted with antibodies against the LIBS-β_3_ domain (ligand-induced binding site), and β_3_ (total integrin β_3_). (C–D) ECs were pretreated with integrin α_v_β_3_ blocking antibody (10 µg/mL) or IgG for 2 h prior to be seeded on HSG and LSG for 24 h followed by qPCR assays for *SEPT9* expression (C) and cell cycle analyses by flow cytometry (D). * p<0.05 in comparison with the corresponding IgG controls on HSG and LSG. § p<0.05 for comparison between IgG control and integrin α_v_β_3_ blocking antibody treatment on HSG. n.s. indicates no significant difference. (n = 5 for *SEPT9* expression and n = 3 for cell cycle analyses).

It has been reported that integrin α_v_β_3_ activates Src family kinases in response to increased matrix-stiffness during tumorigenesis [30] and that cells respond to mechanical tension through the activation of integrin, Rho-GTPases and Src family kinases [31], [32]. To determine the role of integrin α_v_β_3_ in mechanotransduction induced by substrate stiffness, ECs were seeded on HSG and LSG for 24 h, and integrin α_v_β_3_ expression and activation were investigated. Quantification of surface expression of integrins by flow cytometric analyses showed that ECs expressed the same amounts of surface integrin α_v_β_3_ and integrin β_3_ on HSG and LSG (Fig. 6A). However, the use of a monoclonal antibody LIBS-β_3_ that specifically recognizes the active conformation of integrin β_3_ showed that ECs expressed higher levels of activated integrins on HSG than on LSG (Fig. 6B). These results indicate that the activation of integrins, rather than its expression level, may play an important role in regulating EC behavior on substrates with different stiffnesses. Blocking integrin α_v_β_3_ with the antibody LM609 significantly increased the *SEPT9* expression (Fig. 6C), inhibited Src/Vav2 phosphorylations (Fig. S5), and increased G1-phase cells while decreasing S-phase cells on HSG, as compared with control IgG-treatment (Fig. 6D). These results demonstrate that integrin α_v_β_3_ plays a critical role in the regulation of *SEPT9* expression that leads to the stiffness-modulation of cell cycle progression.

## Discussion

In the vascular system, it is believed that the stiffening of the extracellular environment caused by vessel injury promotes cell proliferation and contributes to neointima formation. Studies on *in vivo* mouse vessels have shown that the stiffness is ∼1 kPa for uninjured sites and ∼10 kPa for injured sites - the latter being comparable to the Young's modulus reported for atherosclerotic lesions [24], [25]. Study by Stroka et al. suggests that the range of normal EC substrate stiffness is around 1–4 kPa; the diseased EC substrate stiffness is expected to be greater than 5 kPa and can be up to 280 kPa, which leads to an increase of neutrophil transmigration [5]. EC proliferation rates on glass (>60 GPa), substrates with high-stiffness (21.5 kPa), medium stiffness (9.7 kPa) and lowest stiffness substrates (1.72 kPa) decreased as stiffness decreases (Fig. S1). However, the statistic analyses demonstrated no significant difference between the ECs on glass and substrate of 21.5 kPa, suggesting that 21.5 kPa may have reached the plateau for substrate stiffness-mediated EC proliferation. The 21.5 kPa (HSG) and 1.72 kPa (LSG) substrates (representing the substrate in the range of normal and pathological conditions, respectively) were chosen for the mechanistic studies for the significant differences in their capability in regulating cell function. Our present study has demonstrated that 1) HSG modulates EC proliferation through the activation of integrins/Src/Vav2/RhoA pathway, and 2) LSG inhibits EC proliferation through the inactivation of integrins, leading to an increase of *SEPT9* that attenuates the Src/Vav2/RhoA pathway.

Previous studies have demonstrated that cancer cells and fibroblasts spread well on stiffer substrates with centrally distributed stress fibers and correlate with high proliferation and migration rates [33], [34]. In contrast to stiffer substrates, these cells, when seeded on softer substrates, have lower cellular rigidity and greater cortical actin cytoskeleton distribution [34]. In concert with these findings, our present study showed that ECs on HSG spread well with a central actin stress fiber distribution, whereas ECs on LSG were polygonal in shape with a cortical distribution of actin (Figs. 1A and B). Cell spreading controls many cellular functions through the regulation of RhoA activity [35], and our results also demonstrated that ECs on LSG had lower RhoA activity (Fig. 2A) accompanied by a loss of central actin and an increase of cortical actin distributions. Inhibition of RhoA activity in cells caused the reduction of cell spreading and induction of p27 expression, which led to an attenuation of cell cycle progression [27]. The p27, as a CDK inhibitor, directly inhibits the cyclin-CDK activity to cause the hypophosphorylation of Rb which leads to the down-regulation of cyclin A expression, and consequential growth arrest [36]. Our results also showed that blunted RhoA activity in ECs on LSG led to a decrease of cyclin A, the hypo-phosphorylation of Rb, an increase of p27, and consequent inhibition of EC proliferation. ECs seeded on HSG exhibited opposite results (Figs. 1C and D). Overexpression of the active form of RhoA (RV14) reversed the LSG effects, indicating that RhoA is indeed involved in cell cycle progression in ECs on different substrate stiffness.

In addition to RhoA, the Rho-GTPase family protein Rac has also been reported to regulate the stiffness-mediated cell cycle progression in vascular smooth muscle cells and mouse embryonic fibroblasts through the modulation of cyclin D1 expression [24]. Our results on ECs, however, showed no difference in the expression of cyclin D1 between HSG and LSG; furthermore, overexpression of the active (RacV12) or negative (RacN17) form of Rac had no significant effect on the stiffness-modulated expressions of cell cycle regulatory proteins (Fig. S6). These results suggest that Rac may not be critical for regulating the proliferation of ECs on substrates with different stiffnesses.

Src family kinases have been reported to play important roles in regulating cell functions in response to changes of substrate stiffness [30], [37]. Src has also been shown to be an important molecule that provides the link between integrins and Rho-GTPase signaling pathways through the Vav2 pathway [17]. We found that in comparison to ECs on LSG, ECs on HSG exhibited higher Src activation, which in turn, led to Vav2 phosphorylation at Y172 (Figs. 3A and B). The knockdown of Vav2 expression and treatment with PP1 or Src specific-siRNA to inhibit Src activation or expression, respectively, inhibited RhoA activity, cyclin A expression, Rb hyper-phosphorylation and induced p27 expression in ECs on HSG, thus leading to the consequential EC growth arrest on HSG (Figs 3C, D, E and Fig. S4). These results demonstrate that the stiff substrate can increase EC proliferation via the Src/Vav2/RhoA pathway.

Septins are members of the conserved family of cytoskeletal GTPase proteins and are classified into four groups based on their amino acid similarity [38]. Septins have the intrinsic ability to assemble into polymers and regulate cytoskeleton and membrane organization in mammalian cells [39]. Our results showed a marked induction of *SEPT9* expression in cells on LSG in comparison with the ECs on HSG (Fig. 4A) suggesting that SEPT9 may function as an important regulator for actin organization and RhoA activity in ECs on LSG. Here, we report that SEPT9 gene and protein expressions in ECs on different substrate stiffnesses (Figs. 4A and 5A) were correlated with cortical actin formation (Figs. 1A and 1B). The higher SEPT9 expression in ECs on LSG corresponded to a greater cortical actin distribution than that seen in ECs on HSG (Fig. 1D). The knockdown of SEPT9 in ECs on LSG led to the remodeling of peripheral cortical actin to form central actin fibers (Fig. 4D, upper panels). This observation is quantitatively supported by the intensity plot of the radial distribution of actin (Fig. 4D, lower panel), which shows an extensive loss of the peripheral cortical layer of actin. These results reveal an important role of SEPT9 in the substrate stiffness-modulation of ECs in cortical actin formation. Our results also showed that the knockdown of SEPT9 reversed the down-regulation of RhoA activity on LSG (Fig. 4B). This reversal occurred most likely through the recruitment of p114GEF to directly inhibit RhoA activity (Fig. 5A), as well as through the de-repressive effect on the Src/Vav2 phosphorylations seen in ECs seeded on LSG (Fig. 5B). Src has been reported to regulate Rho activity through activation of GEF and GAP [16]. During initial cell spreading (10–30 mins), Src enhances p190RhoGAP phosphorylation, leading to a down-regulation of Rho activity, whereas at a later spreading stage (40–90 mins), Src increases Rho activity through GEF and promotes actin stress fiber formation [40], [41]. Our results demonstrated that inhibition of SEPT9 expression caused the increase of Src/Vav2 phosphorylations in ECs on LSG (Fig. 5B), suggesting that SEPT9 regulates Src activation and thus exerts an influence on p190GAP. However, the detail role of SEPT9 in modulating Src/p190GAP signaling remains unclear. In addition, when seeded on LSG, ECs transfected with SEPT9-shRNA showed a significant increase in cell proliferation (Figs. 5C and 5D). These findings establish for the first time that SEPT9 acts in a novel role as a regulator responding to substrate stiffness to inhibit RhoA activity on LSG not only by associating with p114GEF but also by inhibiting the Src/Vav2 pathway to attenuate cell cycle progression.

Integrins are important in sensing mechanical cues to regulate cell signaling and functions. ECs express two major fibronectin (FN) receptors: integrin α_v_β_3_ and integrin α_5_β_1_. These two types of integrins are linked to different mechanical functions in the cell. Studies with FN-coated beads have shown that integrin α_5_β_1_ provides a strong adhesive bond that correlates with high force resistance. In contrast, integrin α_v_β_3_ forms unstable bonds with FN that are easily broken by low forces [42]. This force-sensitive integrin α_v_β_3_, instead of the force-resistant integrin α_5_β_1_, may better serve as a sensor for mechanotransduction [43]. In fibroblasts, integrin α_v_β_3_, but not integrin β_1_, mediates the recruitment and activation of Fyn in response to increased matrix stiffness [30]. In addition, integrins β_1_ and β_3_ may play differential roles in modulating RhoA activation. Overexpression of integrin β_3_, but not integrin β_1_ results in an increase in RhoA-GTP levels in cells cultured on FN-coated substrate [44]. Our results demonstrated substrate stiffness-mediated EC proliferation via RhoA signaling pathway. We tested the effects of integrins α_v_β_3_, and β_1_ blocking in ECs on stiffness-mediated RhoA activity. Our results showed that blocking integrin α_v_β_3_ inhibited the HSG-mediated RhoA activation (Fig. S7A). In contrast, blocking integrin β_1_ resulted in no significant change (Fig. S7B). Integrin activation can be controlled by substrate stiffness in many cell types [32], [45], [46]. Moreover, Peng et al. have developed a mathematical model to demonstrate that soft substrates (<2 kPa) produce higher energy barriers for integrin activation and clustering; in contrast, substrates with stiffness ranging from 10 to 100 kPa cause integrin-focal adhesion growth and shorten the time required for firm adhesion [47]. Recent studies on tumors have shown that the ligated/active integrin and unligated/inactive integrin exert opposite actions in regulating cellular functions: the ligated integrin promotes cell proliferation, whereas the unligated integrin inhibits cell proliferation through the transmission of apoptosis signals [48], [49]. Treatment with an integrin α_v_β_3_ antagonist (mimicking the unligated status) inhibited angiogenic vascular cells proliferation in tumor models [50]. In the present study, we observed that while the surface expression levels of integrin α_v_β_3_ and β_3_ on HSG and LSG were similar (Fig. 6A), the activation level of integrin α_v_β_3_ was higher on HSG than that on LSG. Moreover, the integrin α_v_β_3_ antagonist significantly induced the *SEPT9* expression in ECs on HSG (Fig. 6C), suggesting the role of integrin α_v_β_3_ in the differential regulation of SEPT9-mediated EC growth on HSG and LSG.

**Figure 7:**
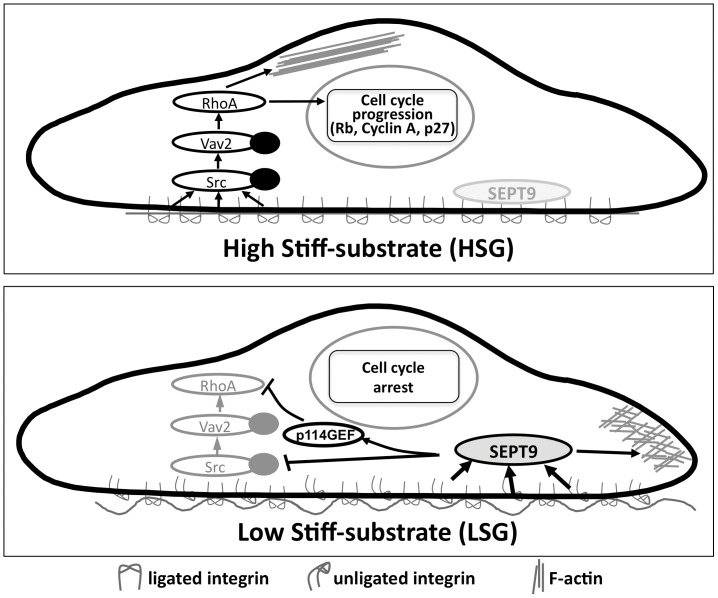
Schematic summary of the mechanisms by which stiffness modulates EC cytoskeleton organization and cell cycle progression.

In summary, our study has established a molecular mechanism by which ECs respond to the alterations of ECM mechanics to regulate their proliferation (Fig. 7). Substrates with high stiffness causes the activation of integrin α_v_β_3_ to inhibit *SEPT9* expression, leading to a central distribution of actin stress fibers, activation of the Src/Vav2/RhoA signaling pathway, and hence EC cell cycle progression. In contrast, substrates with low stiffness causes the inactivation of integrin α_v_β_3_ to increase *SEPT9* expression which induces the peripheral distribution of actin, inhibition of the Src/Vav2/RhoA signaling pathway, and hence repression of EC proliferation. Our findings provide new insights into the mechanism by which ECM mechanics regulate EC proliferation.

## Materials and Methods

### Cell Culture

The Human Umbilical Vein Endothelial Cells (HUVECs) used in this study were isolated from human umbilical cords as previously described [51] and the procedure followed the approved UCSD IRB protocol. HUVECs were cultured in M199 medium (Gibco, Grand Island, NY), supplemented with 10% fetal bovine serum (Gibco), 10% ECGM (Cell Applications, San Diego, CA), 2 mM L-glutamine (Gibco), 1 mM sodium pyruvate, 1% penicillin/streptomycin (Gibco) and maintained in a humidified 5% CO_2_-95% air incubator at 37°C.

### Polyacrylamide Gel Preparation

Gel solutions were prepared by using acrylamide (40% w/v solution, BioRad, Hercules, CA), bis-acrylamide crosslinker (2% w/v solution, Bio-Rad), and 1% HEPES (pH 8.5, EMD Biosciences, San Diego, CA). To prepare gels with different levels of stiffness, mixtures were prepared to contain 5–10% acrylamide and 0.05–0.2% bis-acrylamide and degassed for 20 min to remove oxygen from the solutions. To initiate polymerization, 0.02% APS and 20 µL TEMED were added. A 50–70 µm-thick gel was cast, and the thickness was confirmed through imaging of embedded fluorescent beads in the gel using an Olympus confocal microscope. The results on substrate stiffness determined by atomic force microscopy are shown in Table 1.

Polyacrylamide gels were functionalized to permit cell adhesion by covalently linking the specific ECM ligand (fibronectin) to gel surfaces as previously described [52]. In brief, the gels were activated by exposing the heterobifunctional crosslinker Sulfo-SANPAH (Pierce, Rockford, IL; 0.5 mg/mL in 1×PBS) to UV light. After activation, the gels were washed with 1×PBS to remove excess crosslinker. Gels were then coated with 0.1 mg/mL fibronectin (Sigma, St. Louis, MO) overnight at 4°C or 4 h at room temperature.

### Materials

Rabbit polyclonal antibodies (pAbs) against cyclin A, cyclin D1, p27, phospho-Src and Vav2 were purchased from Santa Cruz Biotechnology (Santa Cruz, CA). Rabbit pAb against hyper-phosphorylated-Rb was purchased from Cell Signaling Technology (Beverly, MA). Mouse mAbs against human α_v_β_3_, β_3_ and β_1_ and LIBS-β_3_ integrins were purchased from Chemicon (Temecula, CA). The control shRNA and specific shRNA of Vav2 were purchased from Santa Cruz Biotechnology. The shRNA of SEPT9 was a kind gift from Dr. K.L. Guan (Department of Pharmacology and Moores Cancer Center, University of California, San Diego). Other chemicals of reagent grade were obtained from Sigma (St. Louis, MO), unless otherwise noted.

### Primer Design

*SEPT7* (sense: 5′-AAGCAAACTGGGAAGCTCAA-3′; antisense: 5′-TCAAACGGATCCAACAAACA-3′; product length, 170 bp)

*SEPT9* (sense: 5′-CCAAGTGACCAGGGAAGTGT-3′; antisense: 5′-AAGGCACGGGTAGATCAACAG-3′; product length, 213 bp).

*SEPT11* (sense: 5′-GAGAAAGCAAATGGGATGGA-3′; antisense: 5′-CACACCTGGCCTGGATTAGT-3′; product length, 202 bp).

### Immunofluorescence Microscopy

Cells were fixed with 2% paraformaldehyde in PBS for 10 min and treated with 1% BSA for 1 h at room temperature. Cells were then treated with FITC-phalloidin for visualizing F-actin and PI for nuclear staining.

### Flow Cytometric Analysis

The harvested cells were fixed for 30 min in cold ethanol (70%), washed, permeabilized with 0.1% Triton X-100 in PBS, stained with 50 mg/mL PI (Invitrogen, Grand Island, NY) and then treated with 1 mg/mL RNase A for 30 min. Stained cells were analyzed with a fluorescence-activated cell sorter (FACS) Calibur (Becton-Dickinson, Franklin Lakes, NJ), and the data were analyzed by using the Flow-Jo cell cycle analysis program. For determination of surface integrin expression, cells were stained with integrin β_3_, integrin α_v_β_3_, and isotype antibodies, and analyzed using the FACS Calibur flow cytometer.

### RNA Isolation and Quantitative qPCR

The total RNA was isolated by using the Trizol reagent and manufacturer protocol. Reverse transcription was carried out with the uses of 2 µg of total RNA and the SuperScript II reverse transcriptase (Invitrogen). The cDNA was amplified through PCR on a 7900HT real-time PCR machine (Applied Biosystems, Foster City, CA) according to manufacturer's protocol. PCR was performed with 0.5 mM primers of Septin genes (i.e., *SEPT7*, *SEPT9* and *SEPT11*) or *Vav2*. Three biological replicates were used for analysis, and all reactions were run in triplicates. The relative levels of gene expressions in ECs were determined using the ΔΔC_T_ method and compared with internal controls.

### Immunoblotting Analysis

The cells were lysed with a buffer containing 1% Nonidet P-40, 0.5% sodium deoxycholate, 0.1% SDS, and a protease inhibitor mixture (PMSF, aprotinin, and sodium orthovanadate). The total cell lysate was separated by SDS/PAGE (12% running, 4% stacking) and non-reducing PAGE for LIBS-β_3_ antibody and transferred onto a nitrocellulose membrane (Immobilon P, 0.45-µm pore size). The membrane was then incubated with the designated antibodies. Immunodetection was performed using the Western-Light chemiluminescent detection system (Applied Biosystems).

### Preparation of Recombinant Proteins

The constitutively active forms of RhoA (RV14) and Rac (RacV12) and the constitutively negative form of Rac (RacN17) were expressed and purified from *Escherichia coli* expression plasmid pGEX4T-1. The GST-tagged RV14, RacV12 and RacN17 were purified from *E. coli* as previously described. The Histidine-tagged C3 exoenzyme was purchased from Cytoskeleton, Inc. (Denver, CO).

### siRNA Delivery Assays

The delivery the SEPT9-shRNA plasmid was carried out by electroporation using a Nucleofector device (Amaxa, Lonza, Swisslands). In brief, cells were resuspended in 100 µl of Nucleofector solution (solution III, Nucleofector kit; Amaxa) together with 5 µg of shRNA plasmid. The cell-plasmid mixture was electroporated using the A034 program on the device. Immediately after electroporation, 400 µl of prewarmed M-199 containing 10% FBS was added and the cells were transferred into culture plates containing 10 ml prewarmed M-199 with 10% FBS. The lentivival Vav2-shRNA was purchased from Santa Cruz Biotech (Santa Cruz, CA). Virus infection was carried out by direct overlay on cells with polybrene (5 µg/ml) following the manufacturer's protocol. Cells were recovered in growth condition for 24 h prior to usage for experiments.

### Immunoprecipitation

The cells were lysed with a buffer containing 25 mM Hepes (pH 7.4), 1% Triton X-100, 1% deoxycholate, 0.1% SDS, 0.125 M NaCl, 5 mM EDTA, 50 mM NaF, 1 mM Na_3_VO_4_, 1 mM PMSF, 10 mg/mL leupeptin, and 2 mM BGP. The cells were disrupted on ice by repeated aspirations through a 21-gauge needle. The same amount of protein from each sample was incubated with a designated antibody for 2 h at 4°C with gentle shaking. The immune complex was then incubated with protein A/G plus agarose for 1 h and collected by centrifugation. The agarose-bound immunoprecipitates were washed and incubated with boiling sample buffer containing 62 mM Tris-HCl (pH 6.7), 1.25% (w/v) SDS, 10% (v/v) glycerol, 3.75% (v/v) mercaptoethanol, and 0.05% (w/v) bromophenol blue. The samples were then subjected to SDS/PAGE and immunoblotting analyses.

### Treatment with mAbs

To block specific integrin-fibronectin interactions, the cells were pre-incubated with an antibody against integrins α_v_β_3_ and β_1_ (10 µg/mL) for 2 h before seeding onto the polyacrylamide gels precoated with fibronectin.

### Statistical analysis

Results are expressed as mean ± SEM. Statistical analysis was performed by using an independent Student t-test for two groups of data and analysis of variance (ANOVA) followed by Scheffe's test for multiple comparisons. A *p* value less than 0.05 was considered statistically significant.

## Supporting Information

Figure S1 **EC proliferation is modulated by stiffnesses of hydrogels in a graded manner.** (A) Young's moduli of hydrogels. The Young's modulus of the hydrogels with differential composition of acrylaminde and Bis were measured by AFM (N = 3). (B) Flow cytometric cell cycle analysis of cells in active DNA synthesizing S phase. This bar graph demonstrated the results of cells in S phase from the BrdU incorporation assay. EC cell cycle analysis derived from flow cytometry data. Cells seeded on glass show the highest percentage of cells in S-phase, while the lowest percentage is present in cells seeded on the softest hydrogel, 1.72 kPa. * P<0.05 when compared to hydrogel 5/0.05. # P<0.05 when compared to glass. Error bars represent SEM.
(PDF)

Figure S2 **Method to measure intensity ratio profile.** F-actin intensities of cells were measured from center to edge of cells. Such radial scans were made for each cell from 0° to 360° for every 10°. Intensities were measured along each radial scan line at 30 points equally distributed between cell center and cell edge (dotted line for the 50° example). All intensity values were normalized to that in the center region (blue region in the schematic with an area = 1% of the cell), which was designated as 1. The minimum intensity value of the F-actin image for the whole cell was subtracted as a background from the measured intensity values before calculation. Cell edges were determined manually from the F-actin staining images.
(PDF)

Figure S3 **RhoA activation contributes to stress fiber formation.** ECs were transfected for 24 h with control or GST-tagged RhoV14 (RV14) or C3 exozyme and then seeded on HSG and LSG for another 24 h. F-actin was stained with FITC-labeled phalloidin. Bar graph showing the F-actin fluorescence intensity values normalized by the cell number in the same microscopic field. (A) RV14 enhanced the F-actin staining on both HSG and LSG, whereas (B) C3 abolished the central F-actin fiber formation on HSG. * *p*<0.05 in comparison with corresponding for comparison controls on HSG and LSG. § *p*<0.05 for comparison between control and RV14 on HSG. # *p*<0.05 between control and RV14 on LSG. (n = 3). Scale Bar = 50 µm.
(PDF)

Figure S4 **Src modulates stiffness-regulated expressions of cell cycle-related proteins.** ECs were transfected with control siRNA (siC, 25 nM) and Src-specific siRNA (siSrc, 25 nM) for 24 h and then were seeded on HSG and LSG for another 24 h. Immunoblotting analyses of cell cycle regulatory proteins were determined by antibodies against hyperphosphorylated Rb, cyclin A, and p27.
(PDF)

Figure S5 **Integrin α_v_β_3_ blocking leads to the attenuation of Src and Vav2 phosphorylations.** ECs were pretreated with integrin α_v_β_3_ blocking antibody (10 µg/ml) or IgG for 2 h prior to be seeded on HSG and LSG for 4 h. Cell lysates were subjected to immunoblotting analyses with antibodies against phospho-Src (Y416), Src, phospho-Vav2 (Y172), and Vav2.
(PDF)

Figure S6 **Rac does not affect stiffness-regulated expressions of cell cycle-related proteins.** ECs were transfected with empty vector, GST-tagged active form of Rac (RacV12), and negative form of Rac (RacN17). After transfection for 24 h, cells were seeded on HSG and LSG for 24 h. Immunoblotting analyses of cell cycle regulatory proteins were determined by antibodies against hyperphosphorylated Rb, cyclin A, cyclin D1, and p27.
(PDF)

Figure S7 **Integrin α_v_β_3_, but not integrin β_1_ involves in HSG-mediated RhoA activation.** ECs were pretreated with integrins α_v_β_3_ and β_1_ blocking antibodies (10 µg/ml) or IgG for 2 h prior to be seeded on HSG and LSG for 4 h. Cell lysates were subjected to an RBD-pull down assay and detected with an antibody against RhoA.
(PDF)
