# Supplementary material for: Matrix Stiffness Regulates Endothelial Cell Proliferation through Septin 9
Source: PLoS One. 2012 Oct 31;7(10):e46889. doi: 10.1371/journal.pone.0046889 (PMC3485289; doi:10.1371/journal.pone.0046889)
Supplement: Figure S1 — EC proliferation is modulated by stiffnesses of hydrogels in a graded manner. (A) Young's moduli of hydrogels. The Young's modulus of the hydrogels with differential composition of acrylaminde and Bis were measured by AFM (N = 3). (B) Flow cytometric cell cycle analysis of cells in active DNA synthesizing S phase. This bar graph demonstrated the results of cells in S phase from the BrdU incorporation assay. EC cell cycle analysis derived from flow cytometry data. Cells seeded on glass show the highest percentage of cells in S-phase, while the lowest percentage is present in cells seeded on the softest hydrogel, 1.72 kPa. * P<0.05 when compared to hydrogel 5/0.05. # P<0.05 when compared to glass. Error bars represent SEM. (PDF) [file pone.0046889.s001.pdf]

Fig. S1

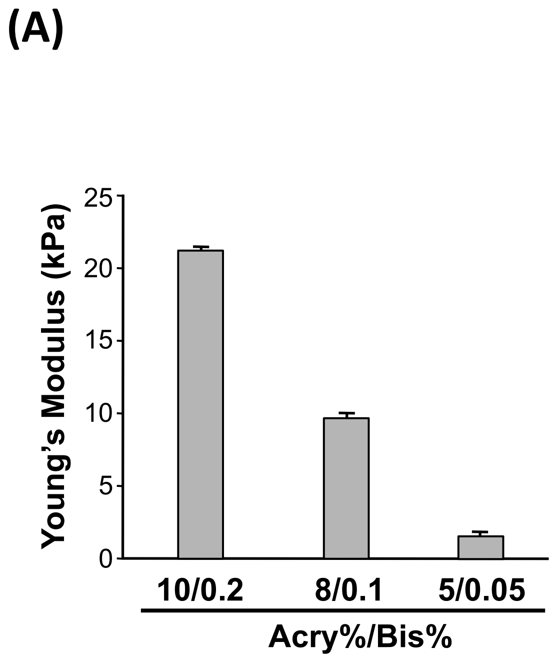

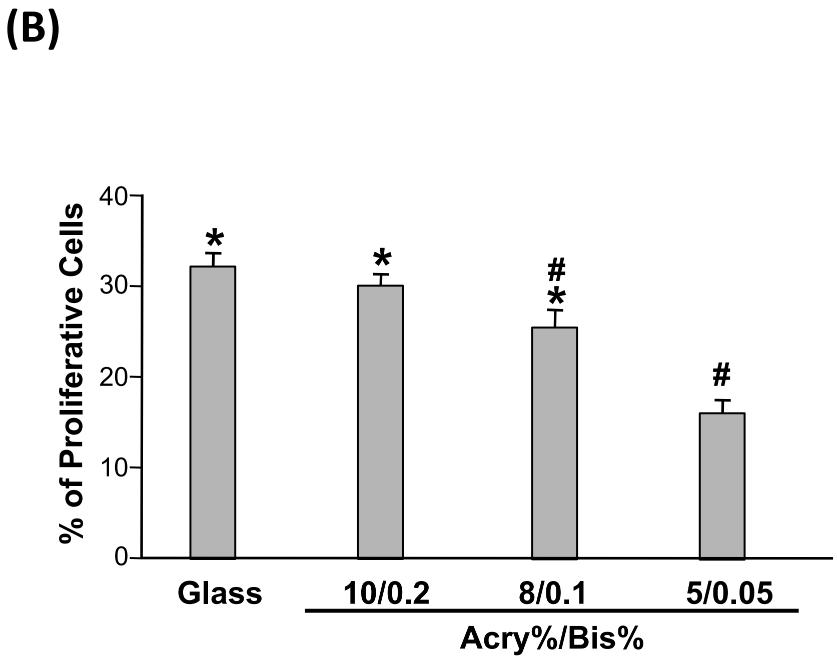

Fig. S1. EC proliferation is modulated by stiffnesses of hydrogels in a graded manner. (A) Young's moduli of hydrogels. The Young's modulus of the hydrogels with differential composition of acrylaminde and Bis were measured by AFM (N=3). (B) Flow cytometric cell cycle analysis of cells in active DNA synthesizing S phase. This bar graph demonstrated the results of cells in S phase from the BrdU incorporation assay. EC cell cycle analysis derived from flow cytometry data. Cells seeded on glass show the highest percentage of cells in S-phase, while the lowest percentage is present in cells seeded on the softest hydrogel, 1.72kPa. * $P < 0.05$ when compared to hydrogel 5/0.05. # $P < 0.05$ when compared to glass. Error bars represent SEM.
