# Supplementary material for: Matrix Stiffness Regulates Endothelial Cell Proliferation through Septin 9
Source: PLoS One. 2012 Oct 31;7(10):e46889. doi: 10.1371/journal.pone.0046889 (PMC3485289; doi:10.1371/journal.pone.0046889)
Supplement: Figure S2 — Method to measure intensity ratio profile. F-actin intensities of cells were measured from center to edge of cells. Such radial scans were made for each cell from 0° to 360° for every 10°. Intensities were measured along each radial scan line at 30 points equally distributed between cell center and cell edge (dotted line for the 50° example). All intensity values were normalized to that in the center region (blue region in the schematic with an area = 1% of the cell), which was designated as 1. The minimum intensity value of the F-actin image for the whole cell was subtracted as a background from the measured intensity values before calculation. Cell edges were determined manually from the F-actin staining images. (PDF) [file pone.0046889.s002.pdf]

Fig. S2

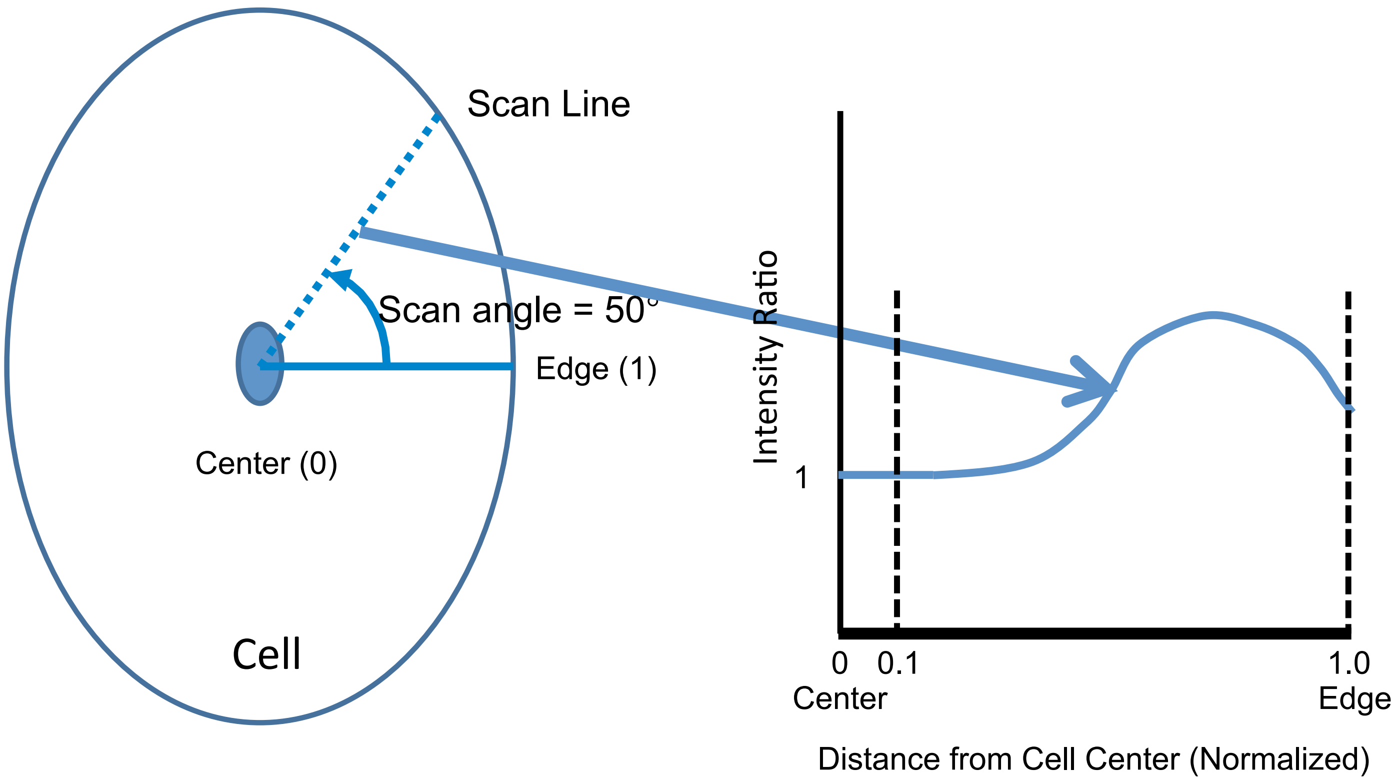

Fig. S2. Method to measure intensity ratio profile. F-actin intensities of cells were measured from center to edge of cells. Such radial scans were made for each cell from 0° to 360° for every 10°. Intensities were measured along each radial scan line at 30 points equally distributed between cell center and cell edge (dotted line for the 50° example). All intensity values were normalized to that in the center region (blue region in the schematic with an area = 1% of the cell), which was designated as 1. The minimum intensity value of the F-actin image for the whole cell was subtracted as a background from the measured intensity values before calculation. Cell edges were determined manually from the F-actin staining images.
