# Supplementary material for: Matrix Stiffness Regulates Endothelial Cell Proliferation through Septin 9
Source: PLoS One. 2012 Oct 31;7(10):e46889. doi: 10.1371/journal.pone.0046889 (PMC3485289; doi:10.1371/journal.pone.0046889)
Supplement: Figure S3 — RhoA activation contributes to stress fiber formation. ECs were transfected for 24 h with control or GST-tagged RhoV14 (RV14) or C3 exozyme and then seeded on HSG and LSG for another 24 h. F-actin was stained with FITC-labeled phalloidin. Bar graph showing the F-actin fluorescence intensity values normalized by the cell number in the same microscopic field. (A) RV14 enhanced the F-actin staining on both HSG and LSG, whereas (B) C3 abolished the central F-actin fiber formation on HSG. * p<0.05 in comparison with corresponding for comparison controls on HSG and LSG. § p<0.05 for comparison between control and RV14 on HSG. # p<0.05 between control and RV14 on LSG. (n = 3). Scale Bar = 50 µm. (PDF) [file pone.0046889.s003.pdf]

Supplementary Fig. S3

(A)

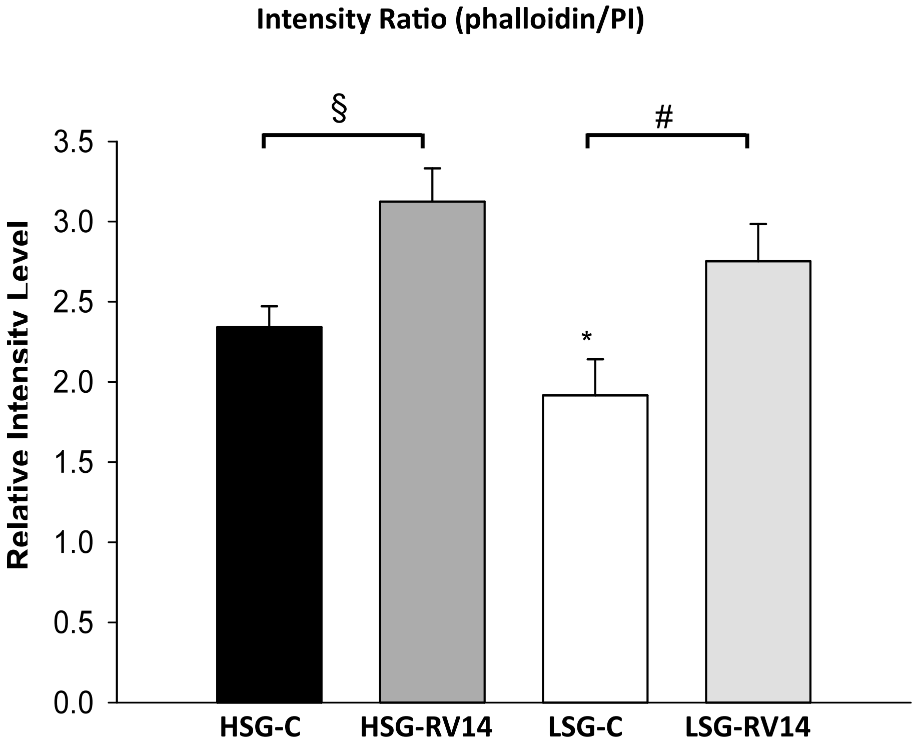

(B)

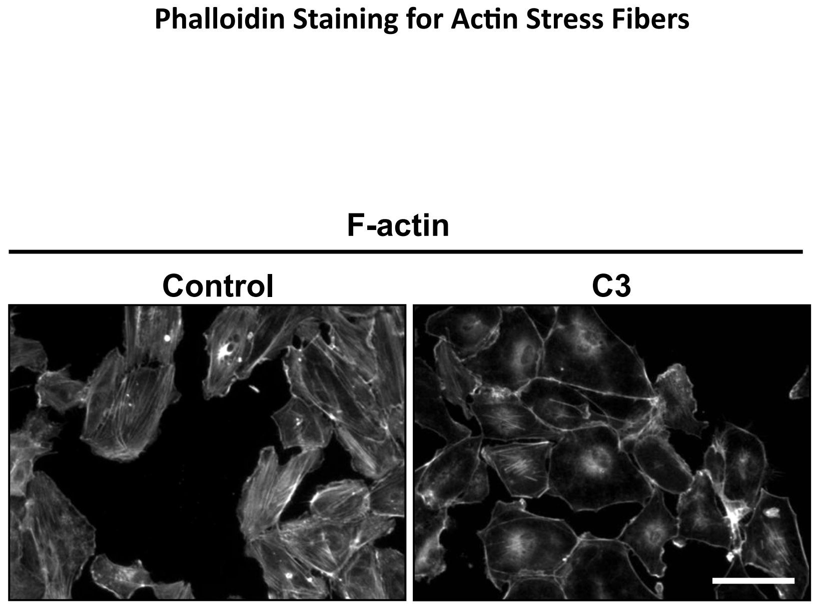

Fig. S3. RhoA activation contributes to stress fiber formation. ECs were transfected for 24 h with control or GST-tagged RhoV14 (RV14) or C3 exozyme and then seeded on HSG and LSG for another 24 h. F-actin was stained with FITC-labeled phalloidin. Bar graph showing the F-actin fluorescence intensity values normalized by the cell number in the same microscopic field. (A) RV14 enhanced the F-actin staining on both HSG and LSG, whereas (B) C3 abolished the central F-actin fiber formation on HSG. * $p<0.05$ in comparison with corresponding for comparison controls on HSG and LSG. § $p<0.05$ for comparison between control and RV14 on HSG. # $p<0.05$ between control and RV14 on LSG. (n = 3). Scale Bar = 50 μm.
