# Supplementary material for: Matrix Stiffness Regulates Endothelial Cell Proliferation through Septin 9
Source: PLoS One. 2012 Oct 31;7(10):e46889. doi: 10.1371/journal.pone.0046889 (PMC3485289; doi:10.1371/journal.pone.0046889)
Supplement: Figure S4 — Src modulates stiffness-regulated expressions of cell cycle-related proteins. ECs were transfected with control siRNA (siC, 25 nM) and Src-specific siRNA (siSrc, 25 nM) for 24 h and then were seeded on HSG and LSG for another 24 h. Immunoblotting analyses of cell cycle regulatory proteins were determined by antibodies against hyperphosphorylated Rb, cyclin A, and p27. (PDF) [file pone.0046889.s004.pdf]

# Fig. S4

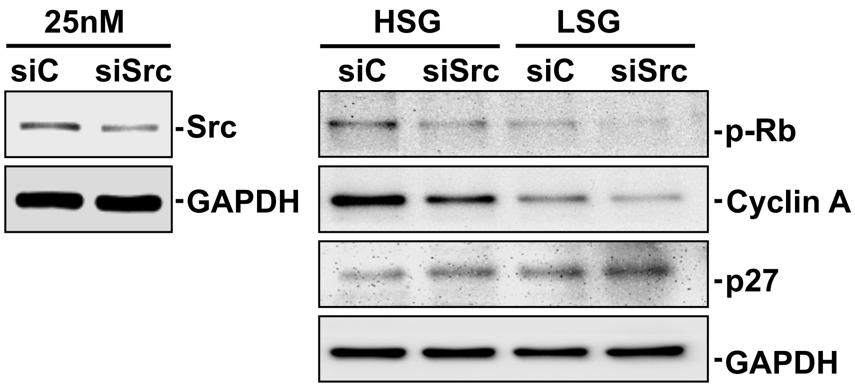


**Fig. S4. Src modulates stiffness-regulated expressions of cell cycle-related proteins.** ECs were transfected with control siRNA (siC, 25nM) and Src-specific siRNA (siSrc, 25nM) for 24 h and then were seeded on HSG and LSG for another 24 h. Immunoblotting analyses of cell cycle regulatory proteins were determined by antibodies against hyperphosphorylated Rb, cyclin A, and p27.
