# Supplementary material for: Matrix Stiffness Regulates Endothelial Cell Proliferation through Septin 9
Source: PLoS One. 2012 Oct 31;7(10):e46889. doi: 10.1371/journal.pone.0046889 (PMC3485289; doi:10.1371/journal.pone.0046889)
Supplement: Figure S5 — Integrin αvβ3 blocking leads to the attenuation of Src and Vav2 phosphorylations. ECs were pretreated with integrin αvβ3 blocking antibody (10 µg/ml) or IgG for 2 h prior to be seeded on HSG and LSG for 4 h. Cell lysates were subjected to immunoblotting analyses with antibodies against phospho-Src (Y416), Src, phospho-Vav2 (Y172), and Vav2. (PDF) [file pone.0046889.s005.pdf]

Fig. S5

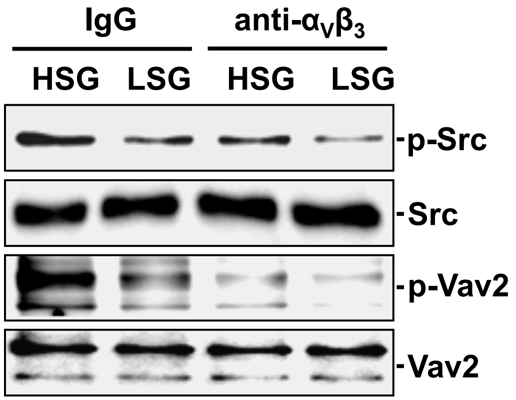

Fig. S5. Integrin $\alpha_v\beta_3$ blocking leads to the attenuation of Src and Vav2 phosphorylations. ECs were pretreated with integrin $\alpha_v\beta_3$ blocking antibody (10 μg/ml) or IgG for 2 h prior to be seeded on HSG and LSG for 4 h. Cell lysates were subjected to immunoblotting analyses with antibodies against phospho-Src (Y416), Src, phospho-Vav2 (Y172), and Vav2.
