# Supplementary material for: Matrix Stiffness Regulates Endothelial Cell Proliferation through Septin 9
Source: PLoS One. 2012 Oct 31;7(10):e46889. doi: 10.1371/journal.pone.0046889 (PMC3485289; doi:10.1371/journal.pone.0046889)
Supplement: Figure S6 — Rac does not affect stiffness-regulated expressions of cell cycle-related proteins. ECs were transfected with empty vector, GST-tagged active form of Rac (RacV12), and negative form of Rac (RacN17). After transfection for 24 h, cells were seeded on HSG and LSG for 24 h. Immunoblotting analyses of cell cycle regulatory proteins were determined by antibodies against hyperphosphorylated Rb, cyclin A, cyclin D1, and p27. (PDF) [file pone.0046889.s006.pdf]

Fig. S6

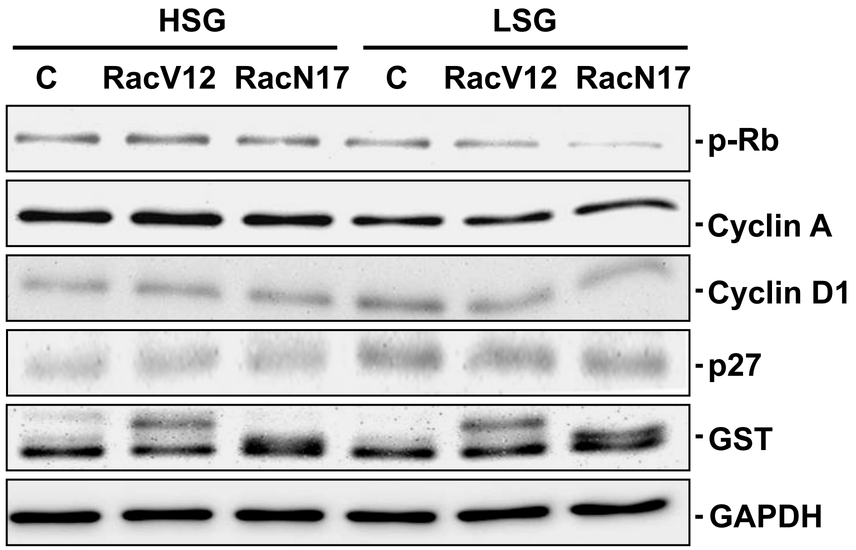

Fig. S6. Rac does not affect stiffness-regulated expressions of cell cycle-related proteins. ECs were transfected with empty vector, GST-tagged active form of Rac (RacV12), and negative form of Rac (RacN17). After transfection for 24 h, cells were seeded on HSG and LSG for 24 h. Immunoblotting analyses of cell cycle regulatory proteins were determined by antibodies against hyperphosphorylated Rb, cyclin A, cyclin D1, and p27.
