# Supplementary material for: Matrix Stiffness Regulates Endothelial Cell Proliferation through Septin 9
Source: PLoS One. 2012 Oct 31;7(10):e46889. doi: 10.1371/journal.pone.0046889 (PMC3485289; doi:10.1371/journal.pone.0046889)
Supplement: Figure S7 — Integrin αvβ3, but not integrin β1 involves in HSG-mediated RhoA activation. ECs were pretreated with integrins αvβ3 and β1 blocking antibodies (10 µg/ml) or IgG for 2 h prior to be seeded on HSG and LSG for 4 h. Cell lysates were subjected to an RBD-pull down assay and detected with an antibody against RhoA. (PDF) [file pone.0046889.s007.pdf]

Fig. S7

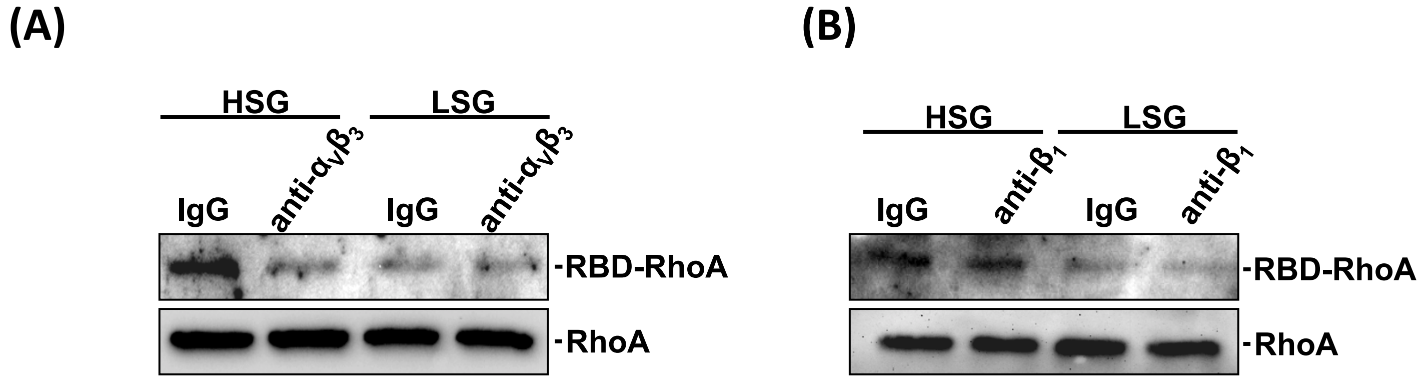

Fig. S7. Integrin $\alpha_v\beta_3$, but not integrin β_1 involves in HSG-mediated RhoA activation. ECs were pretreated with integrins $\alpha_v\beta_3$ and β_1 blocking antibodies (10 μg/ml) or IgG for 2 h prior to be seeded on HSG and LSG for 4 h. Cell lysates were subjected to an RBD-pull down assay and detected with an antibody against RhoA.
